# 15564 Going with the Flow: Engineering Vascularized Urothelial Flaps for Female-to-Male Phalloplasty in Transgender Patients

**DOI:** 10.1017/cts.2021.643

**Published:** 2021-03-31

**Authors:** Jason Harris, Xue Dong, Ryan Bender, Sarah Caughey, Nabih Berri, Jason Spector

**Affiliations:** 1 Weill Cornell Medicine; 2 Downstate Medical Center

## Abstract

ABSTRACT IMPACT: This project uses rapid prototyping, 3D printing, and cell seeding to solve the most common post-operative complications that arise from the current methods of urethral elongation during phalloplasty. OBJECTIVES/GOALS: Post-op fistula and stricture formation occur in up to 50% of phalloplasty patients. These complications arise from the mismatch between skin and uroepithelium, or from scarring secondary to ischemia. Here we describe the fabrication of a novel vascularized urothelial flap for phalloplasty that contains discrete urothelial and vascular channels. METHODS/STUDY POPULATION: A custom designed 3D negative mold, with a urethral channel and a vascular inlet and outlet channel was prototyped in Adobe Fusion 360 and printed on a Prusa i3 MK3S printer in PLA. A 2mm diameter pluronic sacrificial macrofiber was used to connect the channels to form a vascular loop, and 1% type-I collagen was extruded over the mold. After solidifying, the scaffold was demolded and seeded with grade I urothelial carcinoma (SW780 cells, at 5-10 x 106 cells/mL) in the urethral channel, and adenovirus-infected E4 endothelial cells (at 3x106 cells/mL) in the vascular channel. The scaffolds were cultured up to 14 days and then fixed for histologic analysis. RESULTS/ANTICIPATED RESULTS: Collagen scaffolds were fabricated reliably using the custom 3D negative molds. After both seven and fourteen days of culture, the urothelial channel contained a robust, stable urothelial monolayer lining throughout the channel. By 14 days urothelial multilayer formation was seen, providing definitive evidence of a more mature urothelial layer. Grooves within the collagen allowed for nests of urothelial cells to develop, leading to increased multilayer formation. In addition, the vascular channels supported a healthy endothelial lining at both seven and fourteen days. There were no significant histological differences between constructs seeded with 5x106 urothelial cells/mL and 107 cells/mL. We anticipate that multilayer formation will increase with time, and that constructs will survive beyond 28 days. DISCUSSION/SIGNIFICANCE OF FINDINGS: We have developed a novel strategy to engineer vascularized urethral tissue. These constructs can be kept for at least 14 days and form stable monolayers and multilayers consistent with native urothelial architecture. Using 3D printing and autologous cell seeding promises to create patient-specific vascularized urethral flaps for phalloplasty.

